# Measuring critical force in sport climbers: a validation study of the 4 min all-out test on finger flexors

**DOI:** 10.1007/s00421-024-05490-7

**Published:** 2024-04-26

**Authors:** Jiří Baláš, Jan Gajdošík, Tomáš Javorský, Patrik Berta, Andri Feldmann

**Affiliations:** 1 Faculty of Physical Education and Sport, Charles University, José Martího 31, 16252 Prague 6, Czech Republic; 2 Department of Sport Science, University of Innsbruck, Innsbruck, Austria; 3 Institute of Sport Science, University of Bern, Bern, Switzerland

**Keywords:** NIRS, Critical power, Isometric contraction, Muscle oxygen, Intermittent exercise, Threshold

## Abstract

**Purpose:**

The critical force (CF) concept, differentiating steady and non-steady state conditions, extends the critical power paradigm for sport climbing. This study aimed to validate CF for finger flexors derived from the 4 min all-out test as a boundary for the highest sustainable work intensity in sport climbers.

**Methods:**

Twelve participants underwent multiple laboratory visits. Initially, they performed the 4 min intermittent contraction all-out test for CF determination. Subsequent verification visits involved finger-flexor contractions at various intensities, including CF, CF −2 kg, CF −4 kg, and CF −6 kg, lasting for 720 s or until failure, while monitoring muscle-oxygen dynamics of forearm muscles.

**Results:**

CF, determined from the mean force of last three contractions, was measured at 20.1 ± 5.7 kg, while the end-force at 16.8 ± 5.2 kg. In the verification trials, the mean time to failure at CF was 440 ± 140 s, with only one participant completing the 720 s task. When the load was continuously lowered (−2 kg, −4 kg, and −6 kg), a greater number of participants (38%, 69%, and 92%, respectively) successfully completed the 720 s task. Changes of muscle-oxygen dynamics showed a high variability and could not clearly distinguish between exhaustive and non-exhaustive trials.

**Conclusions:**

CF, based on the mean force of the last three contractions, failed to reliably predict the highest sustainable work rate. In contrast, determining CF as the end-force of the last three contractions exhibited a stronger link to sustainable work. Caution is advised in interpreting forearm muscle-oxygen dynamics, lacking sensitivity for nuanced metabolic responses during climbing-related tasks.

**Supplementary Information:**

The online version contains supplementary material available at 10.1007/s00421-024-05490-7.

## Introduction

Sport climbing, a highly technical activity, places significant stress on the forearm flexors. Extensive research has emphasized the critical role of maximal finger-flexor strength and endurance as reliable predictors of climbing ability (Langer et al. 2023; Stien et al. 2022). Notably, research has shown that climbing-specific tests, including both whole-body climbing and isolated finger-flexor tests, demonstrate high predictability for sport climbing performance (Baláš et al. 2021). Various tests involving continuous and intermittent finger-flexor contractions at different intensities have been proposed (Fryer et al. 2015; Limonta et al. 2016; Michailov et al. 2018; Vigouroux and Quaine 2006). Despite efforts to explore the metabolic contributions of sustained and intermittent contractions (Maciejczyk et al. 2022), these tests face limitations in their transferability to prescribing training intensity, as they do not allow for the determination of intensities in metabolic steady and non-steady states.

Understanding maximal exercise intensity steady state is an important threshold concept in exercise performance. The concept of critical power (CP) directly addresses this exercise state defined as the highest exercise intensity that can be sustained for a significant duration of time or physiologically as the highest metabolic rate that can be maintained by supplied oxygen (O_2_) consumption (Poole et al. 2016). Recent developments in climbing research have introduced the concept of critical force (CF) as a metabolic threshold between steady and non-steady state conditions, and an extension of the CP paradigm for sport climbing (Baláš et al. 2022; Giles et al. 2019, 2021). The application of multisession three tasks to failure and the 4 min all-out test has been employed to determine CF on a hangboard (Giles et al. 2019, 2021). In addition, exhaustive climbs at varying wall angles aimed to establish the critical angle as a parallel concept to CP (Baláš et al. 2022). Climbers performing the ascent 2° under critical angle sustained the task for 20 min with moderate perceived exertion, while those climbing 2° above critical angle failed approximately 16 min with higher perceived exertion. Notably, these validations have not been conducted for CF determined from hangboard tests, with only criterion validity against self-reported climbing ability reported. Given the integral role of hangboard training in climbing-specific strength training (Levernier and Laffaye 2019; Medernach et al. 2015), the prescription of appropriate intensity based on a simple diagnostic like the 4 min all-out test becomes crucial. Therefore, the determination of whether CF from the all-out test represents a boundary between localized steady and non-steady conditions is yet to be elucidated.

The 3 min all-out substitute for conventional CP determination was first introduced by Burnley et al. (2006) providing valid estimate of the maximal steady state in cycling. Most participants managed to complete 30 min of exercise at 15 W below the end-test power. In contrast, exercising at 15 W above the end-test power, blood lactate and O_2_ uptake rose inexorably until exhaustion, which occurred in approximately 13 ± 7 min. At a localized level, intermittent handgrip contractions at 15% and 30% under CF from the 5 min all-out test were sustained for 15 min, maintaining an apparent steady state of muscle-O_2_ saturation (StO_2_) (Hammer et al. 2020). Conversely, contractions at 15% and 30% above the CF led to time failure in 602 s and 342 s, respectively, accompanied by a progressive drop in StO_2_ (Hammer et al. 2020). However, conflicting research suggests that the 3 min all-out test either fails to provide accurate estimates of CP (Kalva et al. 2017), with a general tendency to overestimates (Bartram et al. 2017; Karsten et al. 2014). The reason for this discrepancy, likely rooted in the impossibility of sustaining maximal effort for extended periods (Dotan 2022), remains unexplored. Recent discussions ponder whether the CP concept reflects the upper boundary of metabolic steady state (Jones et al. 2019) or if it represents a distinct entity, deviating from the original theory that CP is the highest work rate sustainable without fatigue for an extended duration (Dotan 2022).

The boundary between the heavy and severe exercise intensity domain is considered to represent the transition between sustainable and unsustainable exercise intensity. Various concepts like maximal lactate steady state, respiratory compensation point, deoxy [heme] and StO_2_ localized threshold, and CP serve as markers for this transition. While these concepts share mechanistic principles, their alignment is often lacking. The proposed gray zone (Ozkaya et al. 2022) addresses the gap between commonly used maximal intensity thresholds with CP at the top end and maximal lactate steady state at the bottom end. While these thresholds all are correlated (Caen et al. 2022), they are not interchangeable. To address the boundary between sustainable and unsustainable exercise intensity in climbing-specific conditions, systemic variables (maximal lactate steady state, respiratory compensation point) have not proved valid (Baláš et al. 2021), and assessing localized muscle-O_2_ supply and metabolic O_2_ demands using NIRS (Hammer et al. 2020; Kirby et al. 2021) appears to be the only method to delineate forearm exercise intensity except for CF.

The use of the all-out test to determine CF of finger flexors in climbing is appealing due to its simplicity, however, this test differs from previous forearm all-out tests (Hammer et al. 2020; Kellawan and Tschakovsky 2014) by incorporating longer static contractions in the work:relief cycles and involving upper limb positions overhead, which may limit blood flow and O_2_ delivery. Moreover, during repeated 7 s contractions, fatigue associated with partial vascular occlusion may lead to a continuous drop in applied force within each contraction (Sadamoto et al. 1983), and the mean end-force might not represent the best estimate of CF.

Therefore, the study aimed to verify the concept of CF for finger flexors determined from the 4 min all-out test as a boundary for highest sustainable work intensity in sport climbers. In addition, the study seeks to explore, whether NIRS derived muscle oxygenation is useful in delineating this boundary. We hypothesized that CF, determined as the mean force from the last three contractions of the 4 min all-out test, overestimates the highest sustainable intensity in sport climbers. Furthermore, we expected that muscle-O_2_ dynamics would clearly delineate sustainable and non-sustainable exercise intensities of finger flexors.

## Methods

### Participants

Twelve intermediate to elite sport climbers (10 males and 2 females, 27.2 ± 7.4 years) volunteered to participate in this study. All participants were healthy nonsmokers, not taking any medications, and were required to refrain from strenuous physical activity 24 h before testing and caffeine 12 h prior to testing to prevent any potential ergogenic effects (Guest et al. 2021; Saraiva et al. 2023). They were instructed to maintain consistent activity, diet, and caffeine habits throughout the study. Anthropometric and training characteristics are detailed in Table 1. The study was approved by institutional Ethics Committee in accordance with the Declaration of Helsinki. All participants were informed of the risks of the experiment and signed an informed consent.

**Table 1:** Training and performance characteristics of participants data are reported as means ± SD

Body mass (kg)	68.8 ± 10.8
Height (cm)	176.1 ± 7.7
F _max_ (kg)	53.8 ± 17.0
Climbing experience (years)	11.6 ± 6.8
Climbing ability – lead climbing (IRCRA scale)	19 ± 4
Climbing training (sessions/week)	2.8 ± 1.5
Climbing training (hours/week)	4.9 ± 3.3

F_max_ refers to finger-flexor maximal voluntary contraction of dominant hand. IRCRA scale indicates International Rock Climbing Research Association universal scale for sport climbing ascents

### Design

Participants visited the laboratory five to six times separated by 3–6 days. During their first visit, they completed questionnaires concerning their ability level, climbing preferences and experience and undertook anthropometric measurements (body mass, height). After a warm-up procedure similar to that described by Baláš et al. (2016): 5 min of stair walking, 5 min mobilizing exercises, 5 min traversing on the climbing wall and 5 min individual intermittent hanging on wooden rung), participants completed the test of finger-flexor maximal voluntary contraction (MVC) and 4 min finger-flexor all-out test to determine CF. Subsequent visits involved participants performing finger-flexor contractions at various intensities, including CF, CF −2 kg, CF −4 kg, and CF −6 kg, for 12 min or until failure. An additional visit was conducted with CF −8 kg for a participant unable to sustain CF −6 kg.

### Finger-flexor tests

All finger-flexor tests were conducted using a climbing-specific handgrip dynamometer (1D–SAC, Spacelab, Sofia, Bulgaria). Participants utilized a half-crimp grip position on a 23 mm deep wooden hold with 10 mm radius, excluding the thumb. Tests were performed in a standing position, with the dominant hand and arms at approximately 180° shoulder flexion, elbow slightly flexed. Participants were instructed to progressively transfer their weight (“hang”) on the wooden rung to develop as much force as possible or to attain the target force as quickly as possible. The detail-testing methodology is described elsewhere (Michailov et al. 2018).

### Maximal finger-flexor strength

Maximal finger-flexor strength (in kg) was assessed through the MVC of the finger flexors, involving a 5 s hang on the wooden rung with the dominant hand. The highest peak value from the two trials was considered as maximal finger-flexor strength. The unit kg was used for all finger-strength measurements for easier interpretation of verification trials and comparisons with the original study on CF in climbing (Giles et al. 2021).

### All-out test

The four-minute all-out test was conducted at a work:relief ratio of 7:3 s as proposed by Giles et al. (2021). During the work phase, participants were instructed to produce as much force as possible. During the relief phase, participants were allowed to shake their hand near the body to imitate real sport climbing conditions. In order to evaluate the CF, the mean force derived from the last 30 s of the test was defined (Kellawan and Tschakovsky 2014). Moreover, a decline in force is generally observed during the 7 s contractions. The mean force decrease from the last three contractions was labeled as ∆F_max-min_. In addition, the mean lowest force value from the last three contractions was referred as CF_min_ for further analysis. Impulse from all contractions (W) and impulse over the CF (W’) was calculated to approximate total muscle work and work related to energy store components (Bassan et al. 2019). Rate of force development (RFD) was calculated as the ratio between 50% peak force during a contraction and time to achieve it from the beginning of the contraction. The contraction times during the whole all-out test and last three contractions were computed to identify potential biases.

### Verification of CF trials

To ensure the feasibility and tolerability of exercising at the CF intensity determined from the 4 min all-out test, a series of trials were conducted based on insights gained from previous piloting. Randomly assigned intensities, corresponding to CF or CF −2, −4, −6 kg, were applied during each of the four scheduled visits, all conducted at the same time of day. Participants were instructed to sustain intermittent finger-flexor 7:3 s contractions at the target intensity for 720 s or until failure. Following the completion of each trial, participants promptly provided their rate of perceived exertion (RPE) on a scale ranging from 6 to 20. The selection of a 720 s duration for the test was guided by previous piloting, considering that longer durations often resulted in mechanical discomfort from repetitive contractions. This factor was deemed significant, especially given that such discomfort is not commonly encountered during actual climbing due to frequent changes in grip positions. The highest force that participants were able to tolerate for 720 s was termed as CF_720_.

### Near infrared spectroscopy

**Fig. 1:**
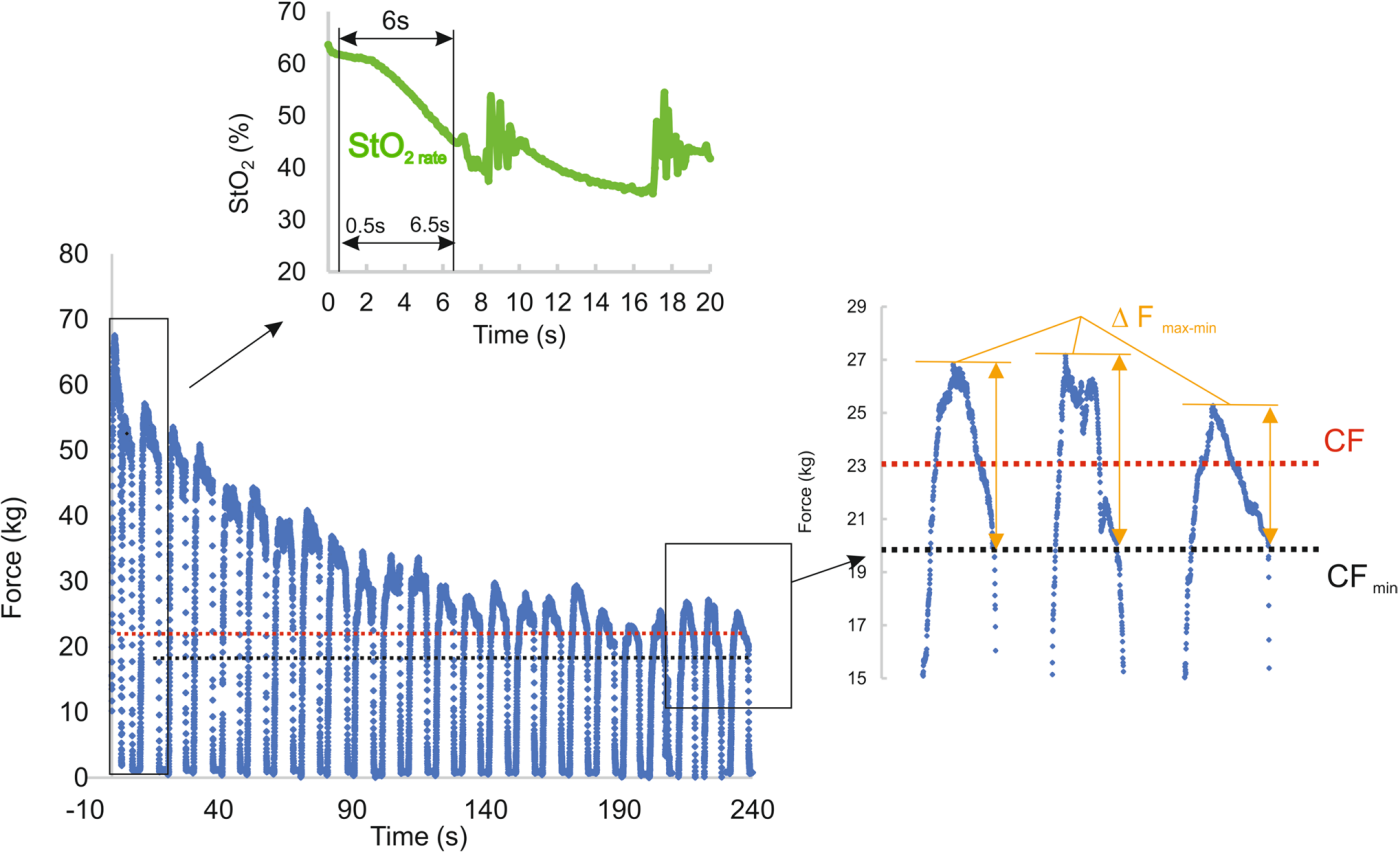
Illustrative example of the all-out test. Red-dashed line represents critical force (CF) determined by the mean force of last three contractions. Black-dashed line represents critical force (CF_min_) determined as the mean of end-force from the last three contractions. The green curve represents muscle-oxygen saturation (StO_2_) changes during the first two contractions and relief cycles. The calculation of the StO_2_ decrease (StO_2 rate_—%/s) was performed using the middle 6 s part of the first 7 s contraction

**Fig. 2:**
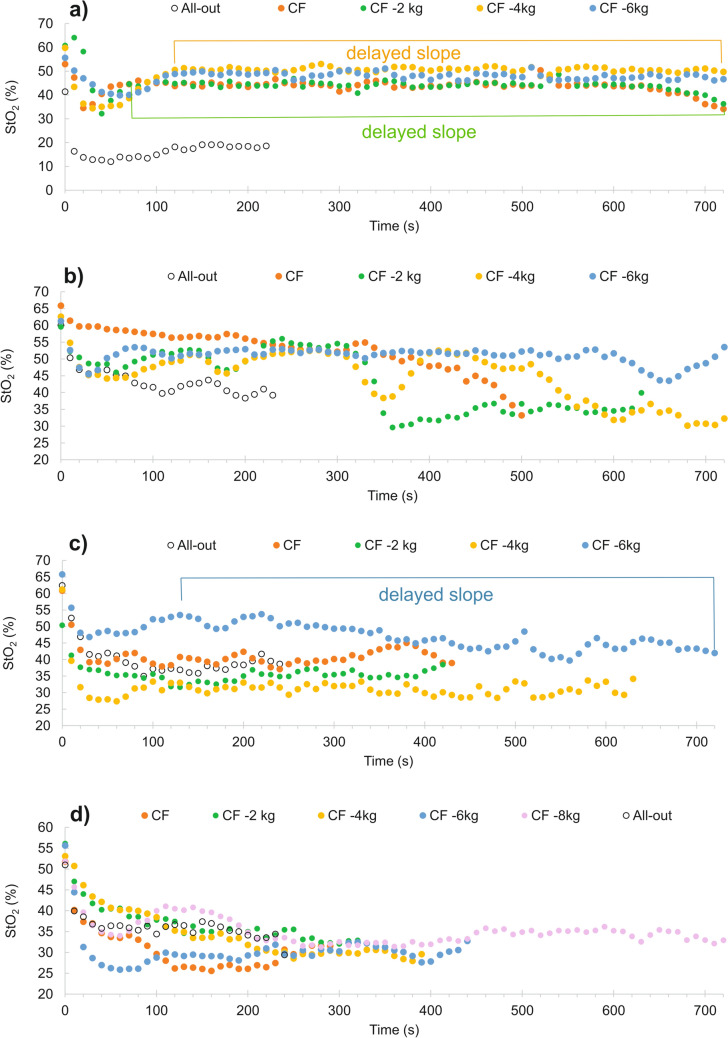
Illustrative examples of muscle-oxygen saturation (StO_2_) from aggregated StO_2_ signal (7 s contraction + 3 s rest) during the all-out tests and verification trials with examples of time periods for delayed slope calculations if the 60 s start-point was not used: **a** the only participant tolerating critical force (CF) for 720 s; **b** participant completing the whole verification trial at the intensity CF −4 kg; **c** participant completing the whole verification trial at the intensity CF-6 kg; **d** the only participant who was able to complete the whole verification trial at the intensity CF −8 kg

Muscle-O_2_ dynamics during all tests were assessed using a continuous wave near infrared spectroscopy (NIRS) device (Portamon, Artinis Medical System, BV, Netherlands). The device was placed over the belly of the flexor digitorum profundus of the dominant hand, following the protocol outlined by Fryer et al. (2018). To minimize optode movement during arm movement and limit ambient light penetration, the device was secured with bi-adhesive tape from the interior and black tape and a black sleeve from the exterior. StO_2_ was used to determine muscle-O_2_ dynamics during all tests. Specific metrics extracted from the all-out test included the rate of StO_2_ decrease during the first contraction (StO_2 rate_), maximal muscle-O_2_ desaturation (StO_2 min_), changes in StO_2_ during relief periods from intermittent contractions during last 30 s (three) contractions (Δ StO_2 relief_) and mean StO_2_ from the last 30 s (StO_2 end_) using 10 Hz sampling frequency. Notably, StO_2 rate_ was computed as % StO_2_ decrease per second between 0.5 and 6.5 s of the first contraction to avoid possible signal noise at the beginning and the end of the contraction (Fig. 1). Data from verification trials were processed using a 10 s moving average (1 work-relief cycle), and parameters such as StO_2_ from the whole test (StO_2 mean_) and last 30 s of the test (StO_2 end_) were analyzed. In addition, the StO_2_-delayed slope (%/min) for the delayed phase after the initial drop in StO_2_ was calculated following the approach proposed by Kirby et al. (2021). Muscle-O_2_ balance, quantified through the StO_2_-delayed slope, establishes a dynamic physiologic threshold distinguishing between sustainable and unsustainable exercise, aligning with the concept of a “critical metabolic rate” and predicts depletion and repletion of finite work capacity and time to exhaustion (Kirby et al. 2021). However, the proposed initial starting time 60 s could not be used universally due longer time of the StO_2_ restoration in some participants and the starting point was determined visually as depicted at Fig. 2. In climbing-specific settings, StO_2_-delayed slope was successfully used to predict the time to exhaustion during sustained finger-flexor contraction (Gilic et al. 2023).

### Statistical analysis

Descriptive statistics (mean ± SD) were used to characterize anthropometric, training, and performance characteristics in all climbers. The Shapiro–Wilk test and visual data inspection using Q–Q plots were conducted to assess data distribution for all variables. In instances where normal distribution was violated, non-parametric approaches were employed for inferential statistics. Differences between mean- and end-time and RFD at the beginning and end of the all-out tests were assessed using Wilcoxon signed-ranked test and paired *t* tests, respectively. The effect of intensity on muscle-O_2_ dynamics (StO_2_-delayed slope, StO_2 mean_, StO_2 end_ during all-out and verification tests was assessed using repeated measure ANOVA with pairwise comparisons employing Bonferroni corrections. The assumption of sphericity was assessed using Mauchly’s test. In cases where the assumption was found to be significant, Huynh–Feldt adjustments were applied. To compare consistency between CF, CF_min_ and CF_720_, intraclass correlation coefficient (ICC) with 95% confidence interval (95%CI), repeated measures analysis of variance and limits of agreement with 95%CI were calculated. The association between climbing ability and selected variables was assessed using Pearson correlation coefficient. More specifically, the relationship between StO_2 rate_, CF and fatigue index was computed using linear regression. Cohen’s *d* for pairwise comparisons and partial eta squared (*µ*_*p*_^2^) were used to assess effect size, statistical significance was set to *P* < 0.05.

## Results

### All-out test

All participants exhibited a plateau of mean force at the end of the all-out test (Fig. 1). CF, determined from the mean force of last three contractions, was measured at 20.1 ± 5.7 kg, representing 38.8 ± 8.8% of maximal finger-flexor strength. A noticeable drop in force during the 7 s contraction attained 8.1 ± 3.2 kg in the last three contractions, leading the CF_min_ to be 3.3 kg lower than CF (Table 2). The time of the contraction at the end of the test did not differ (*P* > 0.05) from the overall mean contraction time ranging around 6.6 s. (Table 2), however a significant decrease of RFD was observed from the start to the end of the test (*P* < 0.001; *d* = 1.65).

**Table 2:** Mean (± SD) force, time, and near infrared spectroscopy variables from the 4 min all-out test

CF – mean force from the last three contractions (kg)	20.1 ± 5.7
W ‘(kg.s)	1365 ± 512
W (kg.s)	4557 ± 1103
Mean contraction time whole test (s)	6.61 ± 0.19
Mean contraction time last three contractions (s)	6.59 ± 0.41
RFD first three contractions (kg/s)	107 ± 42
RFD last three contractions (kg/s)	57 ± 17
CF _min_ – end-force from the last three contraction (kg)	16.8 ± 5.2
CF _720_ (kg)	16.1 ± 4.4
∆ F _max–min_ – mean from last three contractions (kg)	8.1 ± 3.2
StO_2 min_ (%)	28.2 ± 8.7
StO_2 end_ – last three contractions (%)	37.4 ± 8.1
StO_2 rate_ – first contraction (%/s)	2.2 ± 0.9
∆ StO_2 relief_ (%)—last three contractions	11.9 ± 5.7

CF represents the critical force determined as the mean of the last three contractions. W is the impulse from all contractions, W’ is the impulse from all contractions over the CF. RFD refers to the rate of force development. CF_min_ represents the end-force from the last three contractions. CF_720_ was calculated as the highest force that participants were able to tolerate for 720 s

**Fig. 3:**
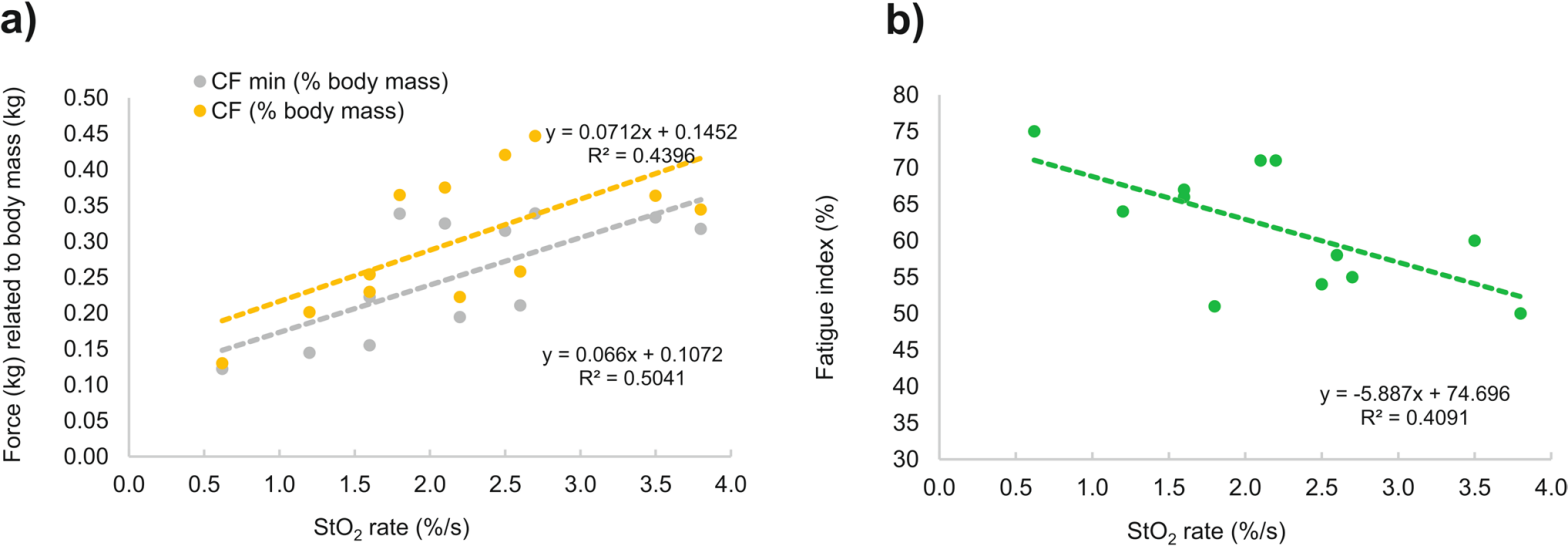
Relationship between muscle oxygen desaturation during the first 7 s contraction of the 4 min all-out test and **a** critical force (CF) normalized to body mass; **b** fatigue index calculated as % of initial force decrease

W and CF normalized to body mass exhibited significant (*P* < 0.05) relationships with climbing ability (*R* = 0.661 for both W and CF). W’ did not reach statistical significance (*P* > 0.05; *R* = 0.552). Notably, a stronger relationship to climbing ability was observed for CF_min_ (*R* = 0.704) and CF_720_ (*R* = 0.699) compared to CF. The StO_2 rate_ explained 44% and 50% of the variability in CF and CF _min_, respectively, and exhibited a moderate correlation with the fatigue index (Fig. 3).

### Verification trials

**Fig. 4:**
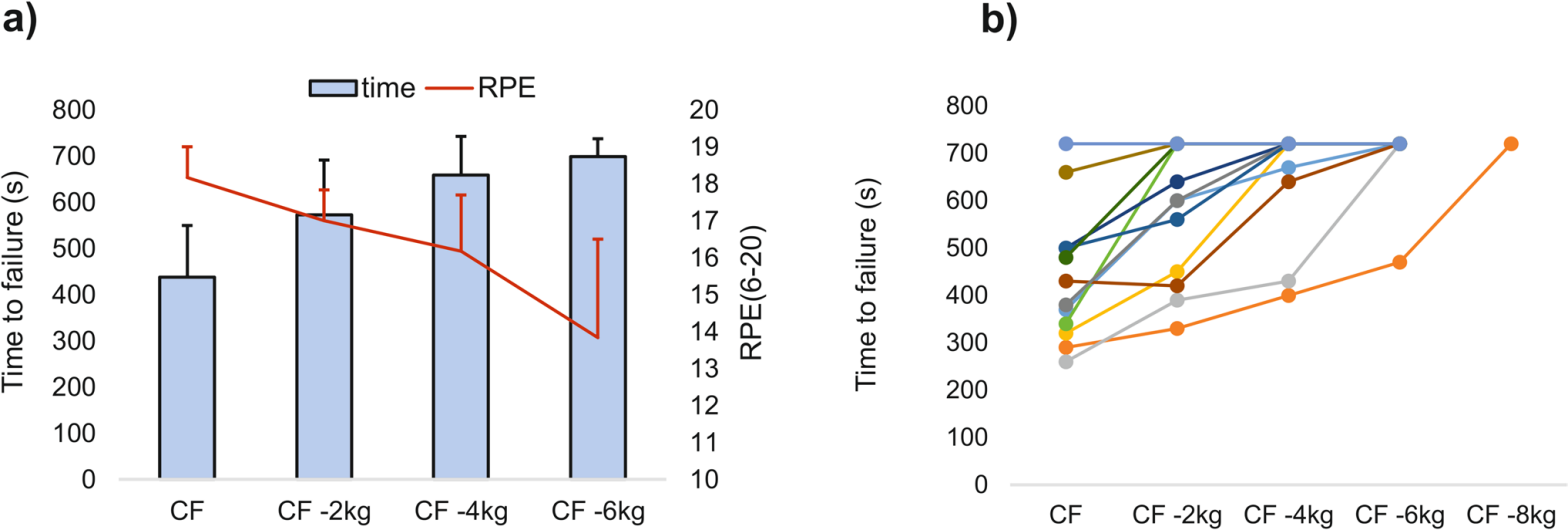
**a** Mean (± SD) time to failure and rate of perceived exertion (RPE) for intensities at critical force (CF), CF-2 kg, CF−4 kg and CF −6 kg; b individual times to failure. Maximum test time was set to 720 s

In the verification trials, the mean time to failure at CF was 440 ± 140 s, with only one participant completing the 720 s task. When the load was continuously lowered (−2 kg, −4 kg, and −6 kg), a greater number of participants (38%, 69%, and 92%, respectively) successfully completed the 720 s task. However, one participant achieved completion only with a load at CF −8 kg (67% of his CF) (Fig. 4).

**Fig. 5:**
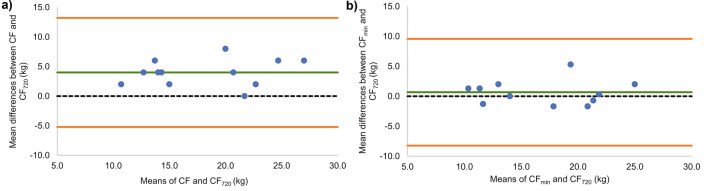
Bland–Altman plots of mean differences (green line) between **a** critical force determined from the mean of the last three all-out test contractions (CF) and minimal force tolerable for 720 s (CF_720_); **b** critical force determined from the end-force of the last three all-out test contractions (CF_min_) and CF_720_. Orange lines represent 95% limits of agreement

CF _720_ was found to be significantly lower (−20%, −4 kg; *P* < 0.001) than CF, with only moderate consistency (ICC = 0.691; 95%CI 0.245 to 0.899) between the two variables. Conversely, no significant differences (0.7 kg; *P* > 0.05) and excellent consistency (ICC = 0.912; 95%CI 0.735 to 0.974) were observed between CF_min_ and CF_720_. The limits of agreement are illustrated at Fig. 5.

**Fig. 6:**
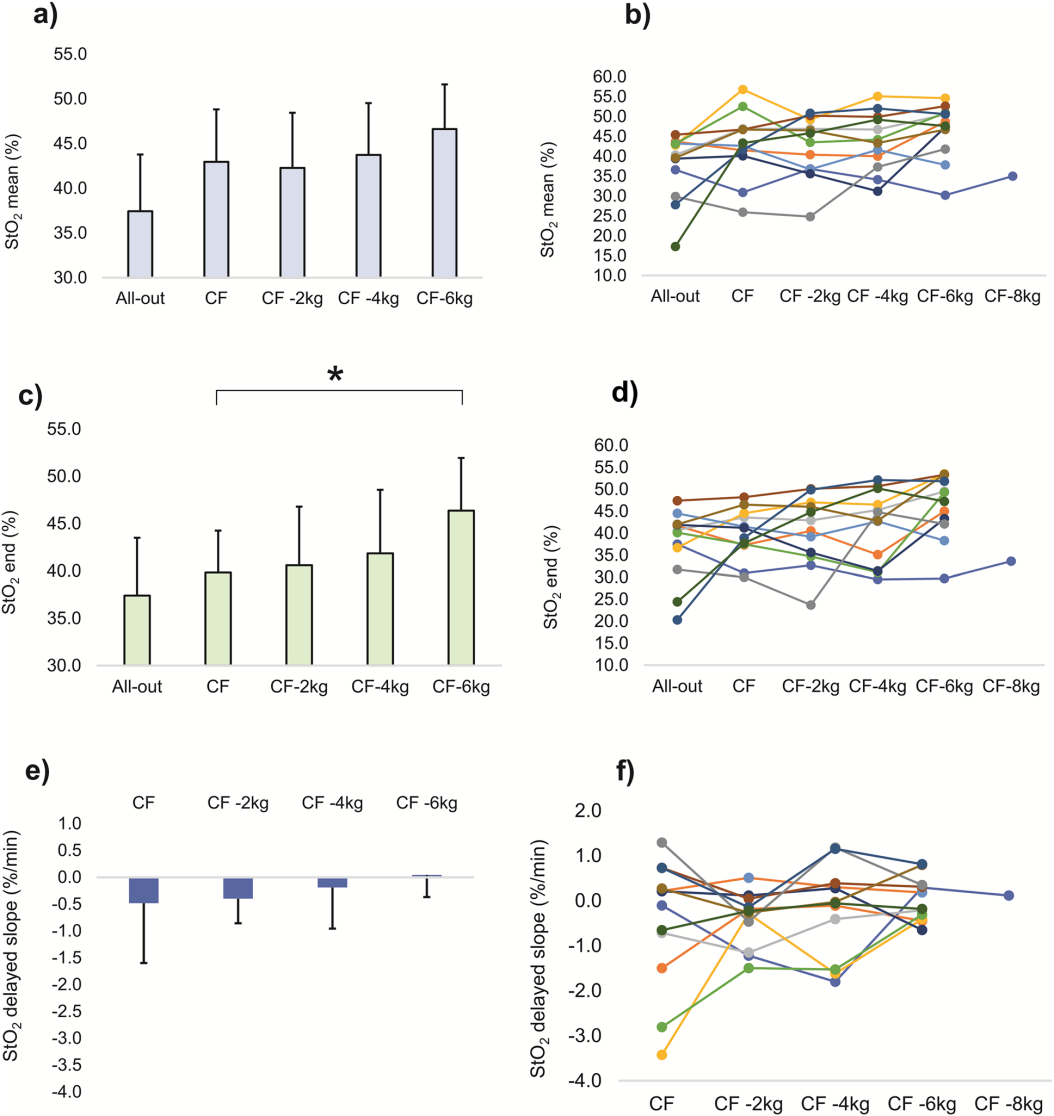
**a** Mean (± SD) and **b** individual muscle-oxygen saturation (StO_2_) from the whole test (StO_2_ mean); **c, d** from the last 30 s (StO_2_ end) of each test; and **e**, **f** delayed StO_2_ slope from all verification trials at the working intensities at the critical force (CF), CF−2 kg, CF−4 kg and CF −6 kg. * indicates significant (*P* < 0.05) differences

The StO_2 end_ significantly increased from all-out test to CF−6 kg trial (*P* = 0.042, *µ*_*p*_^2^ = 0.24). However, pairwise comparisons revealed the only significant difference between CF−6 kg and CF verification trials (6.6%; *P* = 0.011, *d* = 1.01). No other pairwise differences were observed for StO_2 end_ (Fig. 6). In addition, the StO_2 mean_ showed a significant increase from the all-out test to CF−6 kg trial (*P* = 0.006, *µ*_*p*_^2^ = 0.27); however, no pairwise comparisons reached significance (*P* > 0.05) (Fig. 6). Finally, StO_2_-delayed slope did not exhibit statistically significant differences (*P* > 0.05) between verification trials due to high intraindividual variability, although trending to higher values with easier intensity (Fig. 6). In few participants, the StO_2_ development showed irregular drops or increases (Fig. 2b, c), likely due to incontrollable grip and arm positions changes. The mean StO_2 end_ from the all-out was not related to values from the verification trials to failure at CF (−2.4 ± 7.5%; *R* = 0.444; *P* > 0.05) and often, lower StO_2 end_ have been found during verification trials than during the all-out tests (Fig. 2b, c, d). Contrary, in the only participant completing the whole 720 s-long verification trial at CF near exhaustion (RPE = 19), the StO_2 end_ was 13% higher at the exhaustive verification trial than during the all-out test (Fig. 2a).

## Discussion

The study reveals several key findings: 1) CF determined as the mean force from the last three contractions of the 4 min all-out test fails to predict the highest sustainable work rate of forearm flexors; 2) CF_min_ determined as the end-force of the last three contractions shows a stronger correlation with sustainable work rate and better predicts sport climbing ability; 3) the initial-StO_2_ rate during the all-out test is closely related to CF and inversely related to the fatigue index; 4) mean values or delayed slope of StO_2_ are not sensitive enough to discriminate intermittent finger-flexor contractions at intensities ranging from ~ 0–40% below CF in a sport-specific setting.

Despite the widespread use of the 4 min all-out test, our study does not support its predictive validity for sustainable exercise intensity, aligning with current research trends (Bartram et al. 2017; Bergstrom et al. 2013; Karsten et al. 2014). Research indicates that exercises at CP /speed intensity typically last from 15 to 25 min, occasionally extending to 45–60 min (Dotan 2022; Jones et al. 2019). In our study, the mean exercise time to exhaustion at CF was ~ 7 min. However, Jones et al. (2019) discourage the verification of the CP concept by exercising at CP intensity due to potential reliability errors in CP determination. Therefore, we employed four intensities at and below CF, using 2 kg as minimal, practically distinguishing intensity steps to monitor exercise sustainability, RPE, and muscle-O_2_ dynamics. This choice was based on previous reliability data for the forearm flexor 4 min all-out test (Giles et al. 2021; McClean et al. 2023).

Initially, Giles et al. (2021) reported excellent test–retest reliability (ICC = 0.96, limits of agreement = −2.45 to 2.4 kg) for a small sample size. However, a recent study by McClean et al. (2023) with a similar population to ours, revealed lower consistency (ICC = 0.848, CV = 21%) The precision of the test for determining CF was questioned due to significant interindividual variability in the latter study. The only divergence from the initial Giles et al. study (2021) was the allowance of lowering the arm during relief periods, similar to our study, enhancing blood flow, muscle-O_2_ recovery, and performance (Baláš et al. 2016). This modification likely increased CF during the all-out test, however, the effect on reliability is not obvious. The high-interindividual variability in determining CF may be attributed to low hangboarding experience in participants with substantial test–retest CF variability. Familiarization with the exhaustive protocol seems necessary for obtaining reliable data. Although all participants in our study were familiar with the all-out test, we acknowledge the potential reliability error in data interpretation. Considering an ICC of 0.848 and an SD of CF in our sample (SD = 5.7 kg), the standard error of measurement is 2.2 kg, implying that the all-out test may over- or underestimate CF with this value within ~ 68% of the population.

Our results reveal no underestimation of CF. The participant who completed the 720 s CF trial was nearly exhausted at the end. Even considering the reliability error, the intensity of CF −4 kg should be sustainable for nearly all participants, yet only 69% completed the 720 s trial, and the mean RPE was only slightly lower than during the CF trial (18.3 vs 16.3). A significant drop in RPE was observed at CF −6 kg (RPE = 13.8). RPE is generally lower for localized than for whole-body exercise, and research indicates the RPE for the transition between sustainable and non-sustainable climbing to be ~ 14 on the 6–20 scale (Baláš et al. 2022). This suggests that CF, calculated as the mean force from the end of the test, overestimates CF as an indicator of the transition between sustainable and non-sustainable hangboard exercise. Our findings contradict previous forearm studies using the 10 min and 5 min all-out tests to determine CF, which clearly distinguished sustainable and non-sustainable tasks (Hammer et al. 2020; Kellawan and Tschakovsky 2014).

The length of the all-out protocol, particularly the ratio of work-relief cycles, may contribute to these discrepancies. Previous studies utilized short isometric contractions with 1:2 s and 1.5:1.5 s work:relief cycles, respectively (Hammer et al. 2020; Kellawan and Tschakovsky 2014). In such short contractions, there is no force decrease within a contraction, and blood flow restriction is likely smaller compared to longer contraction as showed previously (Broxterman et al. 2014). Partial vascular occlusion begins at ~ 20% of MVC, while full blood occlusion is observed at ~ 50–75% of MVC during handgrip sustained contractions (Barnes 1980; Bergua et al. 2020). Consequently, O_2_ delivery is highly limited during all-out contractions and predominantly occurs in the short relief periods between contractions.

The primary limitation of intermittent hangboard performance appears to be O_2_ delivery rather than O_2_ extraction, as suggested in handgrip exercise with varying work:relief cycles and intensities (Broxterman et al. 2014; Hammer et al. 2020). Enhanced O_2_ extraction likely compensates for reduced O_2_ delivery even during low-intensity exercise, explaining the lack of significant differences in StO_2_ dynamics between all-out tests and verification trials with decreasing intensity in our study. For instance, the StO_2_-delayed slope could not distinguish exhaustive from non-exhaustive trials. Arm movement during relief periods was allowed, and participants, with fatigue, could instinctively change their grip and arm position, affecting forearm flexor involvement (Schweizer and Hudek 2011). These factors are inherent in real climbing conditions and challenging to control even in a standardized setting. Consequently, we observed more or less pronounced waves during the StO_2_-delayed slope (Fig. 2), making the interpretation of StO_2_ data less straightforward. Moreover, the placement of optodes on small forearm muscles may pose an additional challenge compared to voluminous muscles like the vastus lateralis, and even slight rotations of forearm muscles may alter muscle architecture under optodes and StO_2_ responses.

Although StO_2 end_ did not differ between all-out tests and exhaustive verification trials at CF, suggesting that the lowest StO_2_ values can predict exhaustion, caution should be exercised due to large intraindividual variability. This is likely attributed to the low reliability of StO_2 end_ during exhaustive forearm flexor tasks (ICC = 0.437, CV = 23.5%) (Baláš et al. 2018). On the other hand, there was a consistent increase in StO_2 end_ from CF to CF−6 kg trials, reaching statistical and practical significance at CF−6 kg (+ 6.6% StO2 _end_ increase). CF−6 kg might represent a group threshold for sustainable and non-sustainable exercise based on O_2_ dynamics. However, individual increases of StO_2 end_ are not as evident as the whole group means. In summary, due to numerous confounding factors, NIRS may not be sensitive enough to distinguish between steady and non-steady state conditions in climbing-specific conditions at intensities up to 40% below CF.

Another NIRS indicator, the StO_2 rate_ from the beginning of the all-out test, exhibited a close association with CF and an inverse relationship with the fatigue index, calculated as the percentage of force decrease during the test. The rapid decrease in StO_2_ implies a higher rate of muscle-O_2_ extraction, given the absence of changes in blood volume expected during the initial maximal forearm contraction overhead, leading to vascular occlusion. Faster O_2_ extraction is possibly linked to increased mitochondrial respiration capacity, justifying its association with CF, even though it lacks the O_2_ delivery component (Cardinale et al. 2019). Therefore, we propose considering the initial-StO_2_ rate during the all-out test as an additional variable when assessing CF and localized endurance of finger flexors.

The force decrease during each contraction is another factor differing from previous handgrip all-out tests, where no decrease during short contractions was reported (Broxterman et al. 2014; Hammer et al. 2020; Kellawan and Tschakovsky 2014). We observed an ~ 8 kg-force decrease during the last three contractions. Therefore, we tested whether the end-force might better estimate sustainable intensity. It was shown that CF _min_ was in excellent agreement with CF _720_. Moreover, it more precisely predicted lead climbing ability and was more related to StO_2_ rate than CF. All this suggests that the end-force from the last contractions likely more precisely reflects the threshold between sustainable and non-sustainable isometric exercise. However, larger sample size is needed to verify this hypothesis.

RFD significantly decreases during the all-out test, with the initial RFD dropping to half of its values by the end. While this information was not initially part of the objectives and was analyzed to monitor the length and course of each contraction, it is interesting to note. Although not presented, the drop in RFD was not associated with any O_2_ dynamics variables, and due to the low sample size, no assessment of the effect of training status or ability could be performed. Both RFD and force variation during contraction have been linked to specific forearm muscle adaptations in boulder and lead climbers (Fanchini et al. 2013; Limonta et al. 2016). Therefore, these parameters may be further explored to assess neuromuscular fatigue in various ability groups of climbers.

Several limitations and strengths need to be acknowledged. First, the recruited sample size was limited, raising questions about the generalizability of the findings. In addition, testing was conducted throughout different menstrual cycles in two female climbers, which may have contributed to higher variability in strength outcomes (Weidauer et al. 2020). However, within this heterogeneous group of climbers, both time to exhaustion and NIRS responses consistently indicated an overestimation of CF. Furthermore, verification trials employed an absolute load of 2 kg, representing the minimal step, resulting in a slightly varied relative individual intensity under CF for different participants. This pragmatic approach aligns with the feasibility of the study and common practices in hangboard training, where intensity is adjusted by adding or subtracting free weight (Torr et al. 2022). The handgrip contractions were conducted in an ecological setting, enhancing ecological validity while reducing control over all variables (Michailov et al. 2018). This may have contributed to the fluctuation of NIRS signal and reliability of the tests.

## Conclusions

Our study challenges the conventional use of CF derived from the 4 min all-out test for forearm flexors. CF, based on the mean force from the last three contractions, does not predict the highest sustainable work rate reliably.

In contrast, determining CF as the end-force of the last three contractions shows a stronger link to sustainable work rate and better predicts sport climbing ability. Focusing on end-force measurement may provide a more accurate representation of sustained effort capacity in climbing.

The initial-StO_2_ rate during the all-out test correlates closely with CF and inversely with the fatigue index, indicating its potential utility in understanding metabolic demands and fatigue resistance during climbing-specific tasks. However, caution is needed in interpreting mean values or slopes of StO_2_ as discriminatory tools for intermittent finger-flexor contractions around 0–40% below CF in a sport-specific context. Our findings suggest that these conventional metrics may lack the sensitivity required to distinguish nuanced metabolic responses during climbing-related tasks.

## Supplementary Information

Below is the link to the electronic supplementary material.

Supplementary file1 (XLSX 16 KB)

## Data Availability

The datasets generated during in the current study are available as Electronic supplementary material.

## Abbreviations

ANOVA: Analysis of variance
CI: Confidence interval
CF: Critical force calculated as the mean force from the last three contractions
CF_720_: Critical force calculated as the highest force that participants were able to tolerate for 720 s
CF_min_: Critical force calculated as end-force from the last three contractions
CP: Critical power
CV: Coefficient of variation
ICC: Intraclass correlation coefficient
MVC: Maximal voluntary contraction
NIRS: Near-infra red spectroscopy
R: Pearson correlation coefficient
R^2^: Coefficient of determination
RPE: Rate of perceived exertion
O_2_: Oxygen
SD: Standard deviation
StO_2_: Muscle oxygen saturation
W: Impulse from all contractions in the all-out test
W’: Impulse from all contractions in the all-out test over the critical force
RFD: Rate of force development

## References

[CR1] Baláš J, Michailov M, Giles D, Kodejška J, Panáčková M, Fryer S (2016) Active recovery of the finger flexors enhances intermittent handgrip performance in rock climbers. Eur J Sport Sci 16(7):764–772. 10.1080/17461391.2015.1119198 27491378 10.1080/17461391.2015.1119198

[CR2] Baláš J, Kodejška J, Krupková D, Hannsmann J, Fryer S (2018) Reliability of near-infrared spectroscopy for measuring intermittent handgrip contractions in sport climbers. J Strength Cond Res 32(2):494–501 29369955 10.1519/JSC.0000000000002341

[CR3] Baláš J, Gajdošík J, Giles D, Fryer S, Krupková D, Brtník T, Feldmann A (2021) Isolated finger flexor vs. exhaustive whole-body climbing tests? How to assess endurance in sport climbers? Eur J Appl Physiol 121(5):1337–1348. 10.1007/s00421-021-04595-7 33591426 10.1007/s00421-021-04595-7

[CR4] Baláš J, Gajdošík J, Giles D, Fryer S (2022) The estimation of critical angle in climbing as a measure of maximal metabolic steady state. Front Physiol 12:2350 10.3389/fphys.2021.792376 PMC8766676 35069253

[CR5] Barnes WS (1980) The relationship between maximum isometric strength and intramuscular circulatory occlusion. Ergon 23(4):351–357. 10.1080/00140138008924748 10.1080/00140138008924748 7202390

[CR6] Bartram JC, Thewlis D, Martin DT, Norton KI (2017) Predicting critical power in elite cyclists: questioning the validity of the 3 minute all-out test. Int J Sports Physiol Perform 12(6):783–787. 10.1123/ijspp.2016-0376 27834562 10.1123/ijspp.2016-0376

[CR7] Bassan ND, Denadai BS, de Lima LCR, Caritá RAC, Abdalla LHP, Greco CC (2019) Effects of resistance training on impulse above end-test torque and muscle fatigue. Exp Physiol 104(7):1115–1125. 10.1113/ep087204 31131931 10.1113/EP087204

[CR8] Bergstrom HC, Housh TJ, Zuniga JM, Traylor DA, Lewis RW, Camic CL, Johnson GO (2013) Responses during exhaustive exercise at critical power determined from the 3 min all-out test. J Sports Sci 31(5):537–545. 10.1080/02640414.2012.738925 23121405 10.1080/02640414.2012.738925

[CR9] Bergua P, Montero-Marin J, Gomez-Bruton A, Casajús JA (2020) The finger flexors occlusion threshold in sport-climbers: an exploratory study on its indirect approximation. Eur J Sport Sci. 10.1080/17461391.2020.1827047 32962556 10.1080/17461391.2020.1827047

[CR10] Broxterman RM, Ade CJ, Wilcox SL, Schlup SJ, Craig JC, Barstow TJ (2014) Influence of duty cycle on the power-duration relationship: observations and potential mechanisms. Respir Physiol Neurobiol 192:102–111. 10.1016/j.resp.2013.11.010 24361503 10.1016/j.resp.2013.11.010

[CR11] Burnley M, Doust JH, Vanhatalo A (2006) A 3 min all-out test to determine peak oxygen uptake and the maximal steady state. Med Sci Sports Exerc 38(11):1995–2003. 10.1249/01.mss.0000232024.06114.a6 17095935 10.1249/01.mss.0000232024.06114.a6

[CR200] Caen K, Bourgois JG, Stassijns E, Boone J (2022) A longitudinal study on the interchangeable use of whole-body and local exercise thresholds in cycling. Eur J Appl Physiol 122(7):1657–1670. 10.1007/s00421-022-04942-2 35435465 10.1007/s00421-022-04942-2 PMC9014408

[CR12] Cardinale DA, Larsen FJ, Jensen-Urstad M, Rullman E, Sondergaard H, Morales-Alamo D, Boushel R (2019) Muscle mass and inspired oxygen influence oxygen extraction at maximal exercise: role of mitochondrial oxygen affinity. Acta Physiol. 10.1111/apha.13110 10.1111/apha.13110 29863764

[CR13] Dotan R (2022) A critical review of critical power. Eur J Appl Physiol 122(7):1559–1588. 10.1007/s00421-022-04922-6 35303159 10.1007/s00421-022-04922-6

[CR14] Fanchini M, Violette F, Impellizzeri FM, Maffiuletti NA (2013) Differences in climbing-specific strength between boulder and lead rock climbers. J Strength Cond Res 27(2):310–314. 10.1519/JSC.0b013e3182577026 22505133 10.1519/JSC.0b013e3182577026

[CR15] Fryer S, Stoner L, Lucero A, Witter T, Scarrott C, Dickson T, Draper N (2015) Haemodynamic kinetics and intermittent finger flexor performance in rock climbers. Int J Sports Med 36(2):137–142. 10.1055/s-0034-1385887 25251449 10.1055/s-0034-1385887

[CR16] Fryer S, Giles D, Palomino IG, Puerta AD, España-Romero V (2018) Hemodynamic and cardiorespiratory predictors of sport rock climbing performance. J Strength Cond Res 32(12):3534–3541. 10.1519/jsc.0000000000001860 28301444 10.1519/JSC.0000000000001860

[CR17] Giles D, Chidley JB, Taylor N, Torr O, Hadley J, Randall T, Fryer S (2019) The determination of finger-flexor critical force in rock climbers. Int J Sports Physiol Perform 14(7):972–979. 10.1123/ijspp.2018-0809 30676817 10.1123/ijspp.2018-0809

[CR18] Giles D, Hartley C, Maslen H, Hadley J, Taylor N, Torr O, Fryer S (2021) An all-out test to determine finger flexor critical force in rock climbers. Int J Sports Physiol Perform 16(7):942–949. 10.1123/ijspp.2020-0637 33647876 10.1123/ijspp.2020-0637

[CR19] Gilic B, Feldmann A, Vrdoljak D, Sekulic D (2023) Forearm muscle oxygenation and blood volume parameters during sustained contraction performance in youth sport climbers. J Sports Med Phys Fitness 63(7):819–827. 10.23736/s0022-4707.23.14806-7 37154536 10.23736/S0022-4707.23.14806-7

[CR20] Guest NS, VanDusseldorp TA, Nelson MT, Grgic J, Schoenfeld BJ, Jenkins NDM, Campbell BI (2021) International society of sports nutrition position stand: caffeine and exercise performance. J Int Soc of Sports Nutr. 10.1186/s12970-020-00383-4 10.1186/s12970-020-00383-4 PMC7777221 33388079

[CR21] Hammer SM, Alexander AM, Didier KD, Huckaby LM, Barstow TJ (2020) Limb blood flow and muscle oxygenation responses during handgrip exercise above vs. below critical force. Microvasc Res. 10.1016/j.mvr.2020.104002 32198059 10.1016/j.mvr.2020.104002

[CR22] Jones AM, Burnley M, Black MI, Poole DC, Vanhatalo A (2019) The maximal metabolic steady state: redefining the “gold standard.” Physiol Reports. 10.14814/phy2.14098 10.14814/phy2.14098 PMC6533178 31124324

[CR23] Kalva CA, Zagatto AM, da Silva AS, de Araújo MYC, de Almeida PB, Papoti M (2017) Tethered 3-min all-out test did not predict the traditional critical force parameters in inexperienced swimmers. J Sports Med Phys Fitness 57(9):1126–1131. 10.23736/s0022-4707.16.06461-6 27232558 10.23736/S0022-4707.16.06461-6

[CR24] Karsten B, Jobson SA, Hopker J, Passfield L, Beedie C (2014) The 3-min test does not provide a valid measure of critical power using the SRM isokinetic mode. Int J Sports Med 35(4):304–309. 10.1055/s-0033-1349093 24022575 10.1055/s-0033-1349093

[CR25] Kellawan JM, Tschakovsky ME (2014) The single-bout forearm critical force test: a new method to establish forearm aerobic metabolic exercise intensity and capacity. PLoS ONE. 10.1371/journal.pone.0093481 24699366 10.1371/journal.pone.0093481 PMC3974771

[CR26] Kirby BS, Clark DA, Bradley EM, Wilkins BW (2021) The balance of muscle oxygen supply and demand reveals critical metabolic rate and predicts time to exhaustion. J Appl Physiol 130(6):1915–1927. 10.1152/japplphysiol.00058.2021 33914662 10.1152/japplphysiol.00058.2021

[CR27] Langer K, Simon C, Wiemeyer J (2023) Physical performance testing in climbing—a systematic review. Front Sports Act Living. 10.3389/fspor.2023.1130812 37229362 10.3389/fspor.2023.1130812 PMC10203485

[CR28] Levernier G, Laffaye G (2019) Four weeks of finger grip training increases the rate of force development and the maximal force in elite and top world ranking climbers. J Strength Cond Res 33(9):2471–2480. 10.1519/jsc.0000000000002230 28945641 10.1519/JSC.0000000000002230

[CR29] Limonta E, Ce E, Gobbo M, Veicsteinas A, Orizio C, Esposito F (2016) Motor unit activation strategy during a sustained isometric contraction of finger flexor muscles in elite climbers. J Sports Sci 34(2):133–142. 10.1080/02640414.2015.1035738 25897660 10.1080/02640414.2015.1035738

[CR30] Maciejczyk M, Michailov ML, Wiecek M, Szymura J, Rokowski R, Szygula Z, Beneke R (2022) Climbing-specific exercise tests: energy system contributions and relationships with sport performance. Front Physiol. 10.3389/fphys.2021.787902 35140627 10.3389/fphys.2021.787902 PMC8819085

[CR31] McClean ZJ, MacDougall KB, Fletcher JR, Aboodarda SJ, Macintosh BR (2023) Test-retest reliability of a 4 minute all-out critical force test in rock climbers. Int J Exerc Sci 16(4):912–923 37637240 10.70252/DBIC1991 PMC10449326

[CR32] Medernach JPJ, Kleinoder H, Lotzerich HHH (2015) Fingerboard in competitive bouldering: training effects on grip strength and endurance. J Strength Cond Res 29(8):2286–2295. 10.1519/jsc.0000000000000873 26203738 10.1519/JSC.0000000000000873

[CR33] Michailov M, Baláš J, Tanev SK, Andonov HS, Kodejška J, Brown L (2018) Reliability and validity of finger strength and endurance measurements in rock climbing. Res Q Exerc Sport 89(2):246–254. 10.1080/02701367.2018.1441484 29578838 10.1080/02701367.2018.1441484

[CR201] Ozkaya O, Balci GA, As H, Cabuk R, Norouzi M (2022) Grey zone: a gap between heavy and severe exercise domain. J Strength Cond Res 36(1):113–120. 10.1519/jsc.0000000000003427 32149880 10.1519/JSC.0000000000003427

[CR34] Poole DC, Burnley M, Vanhatalo A, Rossiter HB, Jones AM (2016) Critical power: an important fatigue threshold in exercise physiology. Med Sci Sports Exerc 48(11):2320–2334. 10.1249/mss.0000000000000939 27031742 10.1249/MSS.0000000000000939 PMC5070974

[CR35] Sadamoto T, Bondepetersen F, Suzuki Y (1983) Skeletal muscle tension, flow, pressure, and EMG during sustained isometric contractions in humans. Eur J Appl Physiol 51(3):395–408. 10.1007/bf00429076 10.1007/BF00429076 6685038

[CR36] Saraiva SM, Jacinto TA, Gonçalves AC, Gaspar D, Silva LR (2023) Overview of caffeine effects on human health and emerging delivery strategies. Pharm. 10.3390/ph16081067 10.3390/ph16081067 PMC10459237 37630983

[CR37] Schweizer A, Hudek R (2011) Kinetics of Crimp and Slope Grip in Rock Climbing. J Appl Biomech 27(2):116–121 21576719 10.1123/jab.27.2.116

[CR38] Stien N, Saeterbakken AH, Andersen V (2022) Tests and procedures for measuring endurance, strength, and power in climbing-a mini-review. Front Sports and Act Living. 10.3389/fspor.2022.847447 10.3389/fspor.2022.847447 PMC8931302 35308594

[CR39] Torr O, Randall T, Knowles R, Giles D, Atkins S (2022) Reliability and validity of a method for the assessment of sport rock climbers’ isometric finger strength. J Strength Cond Res 36(8):2277–2282. 10.1519/jsc.0000000000003548 32149883 10.1519/JSC.0000000000003548

[CR40] Vigouroux L, Quaine F (2006) Fingertip force and electromyography of finger flexor muscles during a prolonged intermittent exercise in elite climbers and sedentary individuals. J Sports Sci 24(2):181–186 16368628 10.1080/02640410500127785

[CR41] Weidauer L, Zwart MB, Clapper J, Albert J, Vukovich M, Specker B (2020) Neuromuscular performance changes throughout the menstrual cycle in physically active females. J Musculoskelet Neuronal Interact 20(3):314–324 32877968 PMC7493438

