# Correction: Measuring critical force in sport climbers: a validation study of the 4 min all-out test on finger flexors

**DOI:** 10.1007/s00421-024-05658-1

**Published:** 2024-12-12

**Authors:** Jiří Baláš, Jan Gajdošík, Tomáš Javorský, Patrik Berta, Andri Feldmann

**Affiliations:** 1 Faculty of Physical Education and Sport, Charles University, José Martího 31, 16252 Prague 6, Czech Republic; 2 Department of Sport Science, University of Innsbruck, Innsbruck, Austria; 3 Institute of Sport Science, University of Bern, Bern, Switzerland

**Correction: European Journal of Applied Physiology (2024) 124:2787–2798** 10.1007/s00421-024-05490-7

In the original version of this article, the given and family names of author were incorrectly structured. The correct given name and family names should be

Given name: Jiří

Family name: Baláš

Given name: Jan

Family name: Gajdošík

Given name: Tomáš

Family name: Javorský

Given name: Patrik

Family name: Berta

Given name: Andri

Family name: Feldmann

The original article has been corrected.

